# An inside-out origin for the eukaryotic cell

**DOI:** 10.1186/s12915-014-0076-2

**Published:** 2014-10-28

**Authors:** David A Baum, Buzz Baum

**Affiliations:** Department of Botany, University of Wisconsin, 430 Lincoln Drive, Madison, WI 53706 USA; Wisconsin Institute for Discovery, University of Wisconsin, 330 North Orchard Street, Madison, WI 53715 USA; MRC-Laboratory for Molecular Cell Biology, University College London, Gower Street, London, WC1E 6BT UK

**Keywords:** Archaea, Cell topology, Cytoplasmic continuity, ER and endomembrane organization, Evolution of eukaryotes, Mitochondria, Nuclear pore insertion, Origin of the nucleus, Vesicle trafficking

## Abstract

**Background:**

Although the origin of the eukaryotic cell has long been recognized as the single most profound change in cellular organization during the evolution of life on earth, this transition remains poorly understood. Models have always assumed that the nucleus and endomembrane system evolved within the cytoplasm of a prokaryotic cell.

**Results:**

Drawing on diverse aspects of cell biology and phylogenetic data, we invert the traditional interpretation of eukaryotic cell evolution. We propose that an ancestral prokaryotic cell, homologous to the modern-day nucleus, extruded membrane-bound blebs beyond its cell wall. These blebs functioned to facilitate material exchange with ectosymbiotic proto-mitochondria. The cytoplasm was then formed through the expansion of blebs around proto-mitochondria, with continuous spaces between the blebs giving rise to the endoplasmic reticulum, which later evolved into the eukaryotic secretory system. Further bleb-fusion steps yielded a continuous plasma membrane, which served to isolate the endoplasmic reticulum from the environment.

**Conclusions:**

The inside-out theory is consistent with diverse kinds of data and provides an alternative framework by which to explore and understand the dynamic organization of modern eukaryotic cells. It also helps to explain a number of previously enigmatic features of cell biology, including the autonomy of nuclei in syncytia and the subcellular localization of protein N-glycosylation, and makes many predictions, including a novel mechanism of interphase nuclear pore insertion.

## Background

The emergence of the eukaryotic cell with its nucleus, endomembrane system, and membrane-bound organelles represented a quantum leap in complexity beyond anything seen in prokaryotes [1-3]. The sophisticated cellular compartmentalization and the symbiotic association with mitochondria are thought to have enabled eukaryotes to adopt new ecological roles and provided a precursor to numerous successful origins of multicellularity. Nevertheless, despite being recognized as the single most profound evolutionary transition in cellular organization, the origins of the eukaryotic cell remain poorly understood.

The key events in the evolution of eukaryotes were the acquisition of the nucleus, the endomembrane system, and mitochondria. It is now established beyond reasonable doubt that mitochondria are derived from endosymbiotic α-proteobacteria [4-6]. Existing models for the origin of eukaryotes generally agree that proto-mitochondria entered the cell via phagocytosis. Likewise, the most widely favored models for the origins of the nucleus assume that it was formed within a prokaryotic cell as the result of invaginations of the plasma membrane - whether by phagocytosis of an endosymbiont that corresponds to the nuclear compartment or by the internalization of membranes that became organized around the chromatin (reviewed in [7] and discussed further below). Thus, existing theories for the origin of eukaryotes share the assumption that the nucleus is a novel structure formed *within* the boundaries of an existing, and largely unaltered, plasma membrane [8] - they are outside-in models.

Here, we set out to challenge the outside-in perspective. Archaea often generate extracellular protrusions [9-14], but are not known to undergo processes akin to endocytosis or phagocytosis. Therefore, we suggest that eukaryotic cell architecture arose as the result of membrane extrusion. In brief, we propose that eukaryotes evolved from a prokaryotic cell with a single bounding membrane that extended extracellular protrusions that fused to give rise to the cytoplasm and endomembrane system. Under this inside-out model, the nuclear compartment, equivalent to the ancestral prokaryotic cell body, is the oldest part of the cell and remained structurally intact during the transition from prokaryotic to eukaryotic cell organization.

The inside-out model provides a simple stepwise path for the evolution of eukaryotes, which, we argue, fits the existing data at least as well as any current theory. Further, it sheds new light on previously enigmatic features of eukaryotic cell biology, including those that led others to suggest the need to revise current cell theory [15]. Given the large number of testable predictions made by our model, and its potential to stimulate new empirical research, we argue that the inside-out model deserves consideration as a new theory for the origin of eukaryotes.

### Overview of existing models of eukaryotic cell evolution

Endosymbiotic, outside-in models explain the origin of the nucleus and mitochondria as being the result of sequential rounds of phagocytosis and endosymbiosis. These models invoke three partners - host, nucleus, and mitochondria - and envisage the nuclear compartment being derived from an endosymbiont that was engulfed by a host cell. Authors have suggested that the host (that is, cytoplasm) could be an archaeon [16-18], a proteobacterium [19-21], or a bacterium of the Planctomycetes, Verrucomicrobia, Chlamydiae (PVC) superphylum [22]. The endosymbiont (that is, the nucleus) has been proposed to have been an archaeon [19-22], a spirochete [16], or a membrane-bound virus [17,18]. In general, endosymbiotic models are agnostic as to whether mitochondria were acquired before or after the nucleus. An exception to this is the syntrophic consortium model, which envisages the simultaneous fusion of a symbiotic community composed of all three partners: cytoplasm, nucleus, and mitochondria [23,24]. A more divergent ‘endosymbiotic’ model is the endospore model [25]. This holds that the nucleus evolved when a cell enclosed its sister after cell division, similar to the way in which endospores are formed in certain Gram-positive bacteria. However, there is no evidence of endospore formation or other engulfment processes in Archaea, making this hypothesis improbable.

Recent phylogenomic analyses have revealed that the eukaryotic genome likely represents a combination of two genomes, one archaeal [26,27] and one proteobacterial [28,29]. There is no evidence to support any additional, major genome donor as expected under nuclear endosymbiotic models [30]. Furthermore, endosymbiotic models (including the endospore model) require supplemental theories to explain the origin of the endomembrane system, the physical continuity of inner and outer nuclear membranes, and the formation of nuclear pores. In light of these facts, we do not think that endosymbiosis provides a convincing explanation for the origin of the nuclear compartment [2,7,31-33].

Given the problems with endosymbiotic models, we believe that the most compelling current models for the origin of eukaryotes are those that invoke an autogenous origin of the nucleus. These usually suggest that a prokaryotic ancestor evolved the ability to invaginate membranes to generate internal membrane-bound compartments, which became organized around chromatin to generate a nucleus [32,34-36]. In some models, infoldings of the plasma membrane were pinched off to form endoplasmic reticulum (ER)-like internal compartments that later became organized around the chromatin to form the inner and outer nuclear envelope [35,37-39]. Alternatively, the nuclear membranes could be seen as arising from invaginations of the plasma membrane, so that the early eukaryote cell had an ER and nuclear envelope that were continuous with the outer cell membrane [40]. In either case, under these models the nuclear membrane is ultimately derived from internalized plasma membrane.

Older autogenous outside-in models generally proposed that mitochondria were acquired by a cell that already had a nucleus [32,34,35] - in line with the results of early phylogenetic studies [41]. More recent phylogenetic data have suggested that mitochondria were present in the last eukaryotic common ancestor [42,43]. This has led to the formulation of new autogenous models in which the acquisition of mitochondria predates the formation of the nuclear compartment [1,23,44-46].

### Overview of the inside-out model

**Figure 1:**
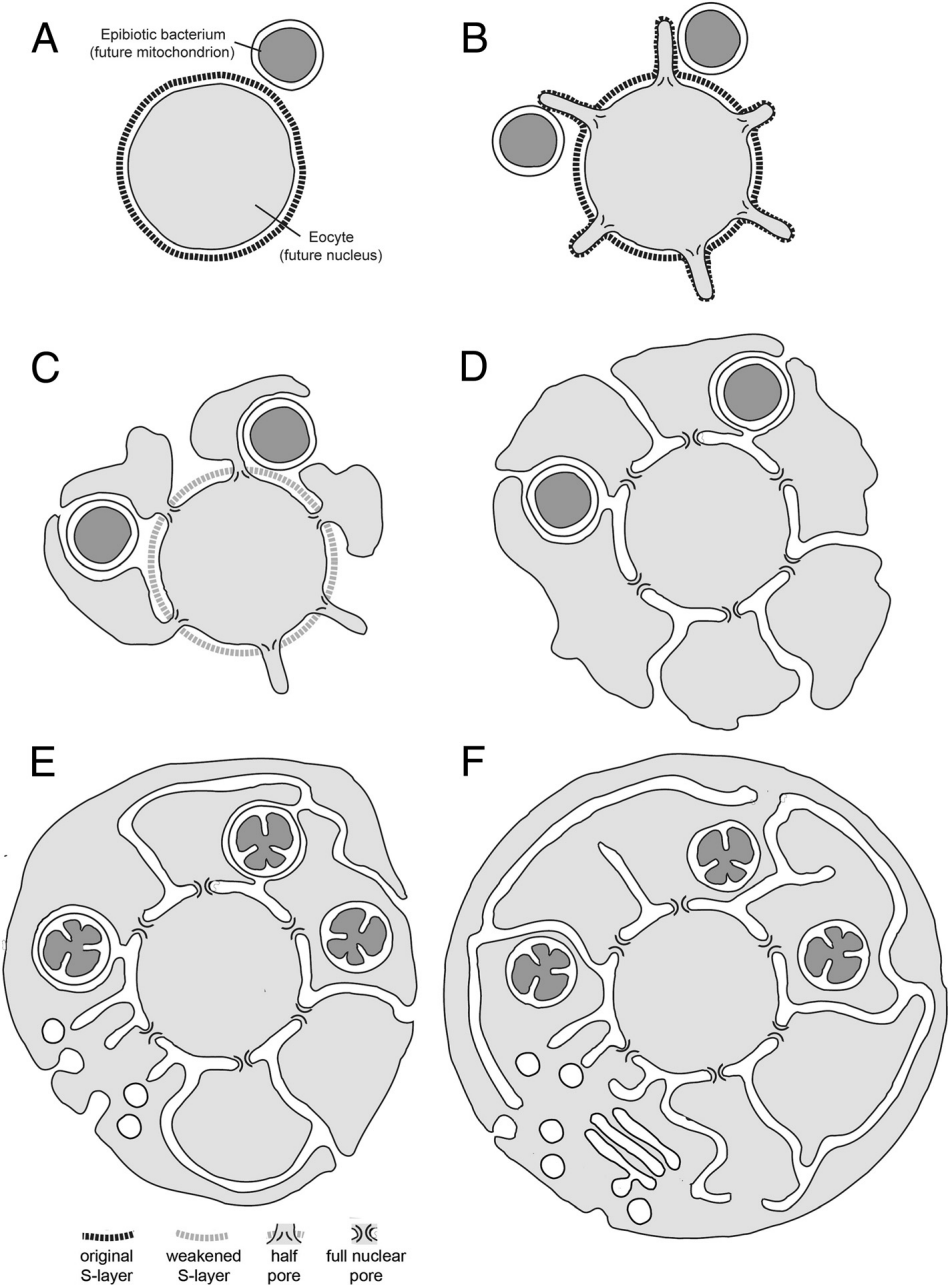
**Inside-out model for the evolution of eukaryotic cell organization.** Model showing the stepwise evolution of eukaryotic cell organization from **(A)** an eocyte ancestor with a single bounding membrane and a glycoprotein rich cell wall (S-layer) interacting with epibiotic α-proteobacteria (proto-mitochondria). **(B)** We envision the eocyte cell forming protrusions, aided by protein-membrane interactions at the protrusion neck. These protrusions facilitated material exchange with proto-mitochondria. **(C)** Selection for a greater area of contact between the symbionts would have led to bleb enlargement and the eventual loss of the S-layer from the protrusions. **(D)** Blebs would have then been further stabilized by the development of a symmetric nuclear pore outer ring complex (Figure 2) and through the establishment of LINC complexes that, following the gradual loss of the S-layer, physically connected the original cell body (the nascent nuclear compartment) to the inner bleb membranes. **(E)** With the expansion of blebs to enclose the proto-mitochondria, a process that would have facilitated the acquisition of bacterial lipid biosynthesis machinery by the host, the site of cell growth would have progressively shifted to the cytoplasm, facilitated by the development of regulated traffic through the nuclear pore. At the same time, the spaces between blebs would have enabled the gradual maturation of proteins secreted into the environment via the perinuclear space through glycosylation and proteolytic cleavage. **(F)** Finally, bleb fusion would have connected cytoplasmic compartments and driven the formation of an intact plasma membrane, perhaps through a process akin to phagocytosis whereby one bleb enveloped the whole. This simple topological transition would have isolated the endoplasmic reticulum from the outside world, driven the full development of a system of vesicular trafficking, and established strict vertical transmission of mitochondria, leading to a cell with modern eukaryotic cell organization.

In following sections we outline a series of simple evolutionary steps from a prokaryotic to a fully eukaryotic cell structure, driven primarily by selection for an increasingly intimate mutualistic association between an archaeal host cell and α-proteobacteria (proto-mitochondria), which initially lived on the host cell surface (Figure 1). Under the inside-out hypothesis, the outer nuclear membrane, plasma membrane, and cytoplasm were derived from extracellular protrusions (blebs), whereas the ER represents the spaces between blebs (Table 1). Mitochondria were initially trapped in the ER, but later penetrated the ER membrane to enter the cytoplasm proper. Under the inside-out model, the final step in eukaryogenesis was the formation of a continuous plasma membrane, which closed off the ER from the exterior.

**Table 1:** **Homologies under three competing models for the origin of eukaryotes**

**Eukaryotic cell structure**	**Autogenous outside-in**	**Endosymbiotic outside-in**	**Inside-out**
Inner nuclear membrane	Inner surface of fused ER lamellae	Plasma membrane of endosymbiont	Original plasma membrane
Perinuclear space	ER cisterna	Food vacuole	Footprint of the original cell wall
Outer nuclear membrane	Outer surface of fused ER lamellae	Food vacuole membrane	Inner surface of cytoplasmic blebs
ER	Internalized plasma membrane vesicles	Internalized plasma membrane vesicles	Space between extracellular blebs
Plasma membrane	Original plasma membrane	Original plasma membrane	Outer membrane of extracellular blebs

ER, endoplasmic reticulum.

Only one other paper that we are aware of has proposed that the nuclear compartment corresponds to boundaries of an ancestral cell. The exomembrane hypothesis of de Roos [47] is, however, quite distinct from the model put forward here. De Roos postulated that the starting point was a proto-eukaryote with a double membrane that secreted membranous extracellular vesicles that fused to form an enclosing plasma membrane. Moreover, his model relies on an unconventional view of evolutionary history, including an independent origin of eukaryotic and prokaryotic cells. Thus, we will not discuss the exomembrane hypothesis further.

In the following sections, we describe the inside-out model in detail. We discuss the cellular processes involved in the generation of the cytoplasmic compartment, the vesicle trafficking system and plasma membrane, and cilia and flagella. In each section we point to relevant selective drivers and supporting evidence. Finally, we look at some of the implications and testable predictions of the model and conclude by reflecting on the prospects for determining which of the models, inside-out or outside-in, is more likely to be correct.

## Results and Discussion

### Extracellular protrusions arose to facilitate material exchange with the external environment

We take as our starting point a prokaryotic cell similar to an ‘eocyte’ [48], an informal name that has come to refer to a member of the archaeal phyla Crenarchaeota, Thaumarchaeota, and Korarchaeota [49]. Eocytes usually have a single lipid bilayer membrane and a simple cell wall (S-layer) rich in N-glycosylated proteins [50]. They also have a relatively well-developed cytoskeleton that includes homologs of actin and tubulin [51-53] and the membrane-manipulating protein ESCRTIII [54-58].

Recent phylogenetic studies have tended to support the ‘eocyte hypothesis,’ which holds that eocytes are more closely related to eukaryotes than they are to euryarchaeote Archaea [26,27,48,59,60], though this conclusion is disputed [61]. While the inside-out hypothesis is not formally dependent on the veracity of the eocyte hypothesis, as we show below, the eocyte hypothesis poses a significant challenge to any outside-in hypothesis proposed to date.

Under the inside-out model, the pre-eukaryote developed outward protrusions (Figure 1A,B). Many Archaea, including some eocytes [11,13,62], exhibit such structures [9-14,62], but they are rarely seen in bacteria [54,63]. In almost all cases where the images are clear, protrusions are bounded by an S-layer. In some living Archaea, ESCRTIII has been inferred to pinch off protrusions to yield extracellular membrane vesicles [54,55]. However, if scission were suppressed, long-lived protrusions could be formed.

The stable protrusions formed by suppression of scission would have increased the surface-to-volume ratio of the host cell. The idea that an eocyte might produce extracellular protrusions as a means to increase its surface area is justified by the observation that protrusion formation is stimulated in the crenarchaeote *Stettaria hydrogenophila* in response to reductions in the concentration of extracellular sulfur [9]. Moreover, Archaea with protrusions associated with cell-cell contacts have been seen in mixed microbial communities in biofilms [12].

The potential selective value of extracellular protrusions is also illustrated by a number of living eukaryotic groups, such as foraminiferans and radiolarians, which have a central cell body enclosed within a rigid test that has pores through which protrusions project. This arrangement allows cells to interact directly and dynamically with the external environment while retaining their genetic material in a protective keep. These phyla are ecologically successful, with many thousands of living and extinct species [64]. The rapid radiation of foraminiferans in the Cambrian, not long after the evolution of rigid tests [65], makes clear the potential advantages of a cell increasing its surface area while retaining its chromatin in a protective inner compartment. Further, it is noteworthy that in some rhizarian subgroups, pseudopodia fuse with one another to generate an extra-testal compartment that is loosely analogous to a continuous cytoplasm forming via the fusion of extracellular blebs.

### The molecular machinery underlying the formation of stable protrusions

Little is currently known about the cell biology of archaeal protrusions. Specifically, it is unclear how protrusions are formed and stabilized. This could be achieved through the action of proteins at the protrusion neck, by an internal cytoskeleton, by structural changes in the S-layer (for example, local weakening), by changes in the connections between the S-layer and cytoplasmic proteins, and/or by changes in osmotic pressure. How cells generate stable protrusions is important for the model, since this corresponds to the first step in the evolution of the cytoplasm (Figure 1).

We speculate that positive curvature at the protrusion’s base was first stabilized by proteins containing seven-blade β-propeller domains homologous to Coat Protein II (COPII)-like proteins that form the outer ring of the nuclear pore complex (NPC) (Figures 1B,C). Many proteins with seven-blade β-propeller domains are found in prokaryotes, including some that are localized to the periphery of living archaea [66]. It is not yet known whether these or other prokaryotic β-propeller domain proteins are direct homologs of NPC proteins. COPII-like proteins do not associate with membranes directly, but interact with membranes via diverse membrane-binding proteins [67,68]. Nonetheless, they play a conserved role in stabilizing positive membrane curvature [69], making them a natural candidate for having an ancestral role in stabilizing the bases of extracellular protrusions - a cellular location that corresponds to the nuclear pore of modern eukaryotes.

**Figure 2:**
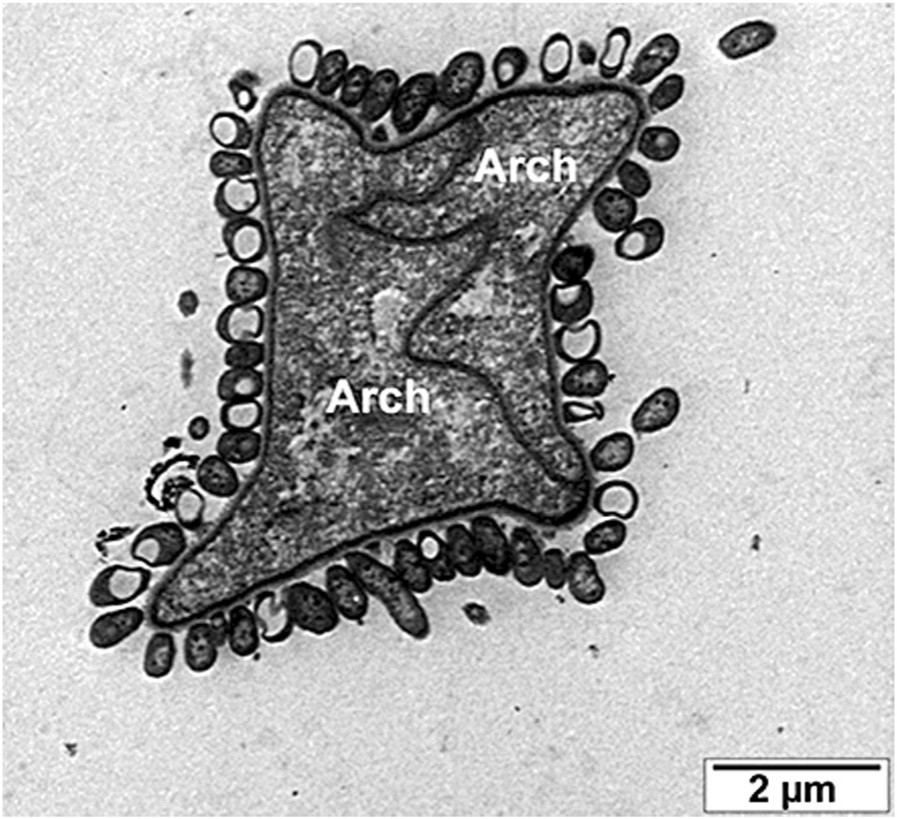
**Example of epibiotic bacteria associated with archaeal cells.** Image of two Candidatus *Giganthauma karukerense* cells surrounded by ectosymbiotic γ-proteobacteria (reproduced with permission from [84]).

It has been claimed that homologs of α-solenoid domains, which occur in many nucleoporins, are common in prokaryotes, and are coupled to β-propeller domains in some PVC bacteria [70]. However, these appear to be generic α-helical repeat domains rather than true homologs of nucleoporin α-solenoid domains [71]. Therefore, in the absence of more concrete data, we hypothesize that α-solenoid and β-propeller domains came together in a single protein in the eocyte ancestor of eukaryotes and that this fused ancestral protein gave rise, via gene duplication, to the outer-ring nucleoporins of the modern NPC [72-74].

**Figure 3:**
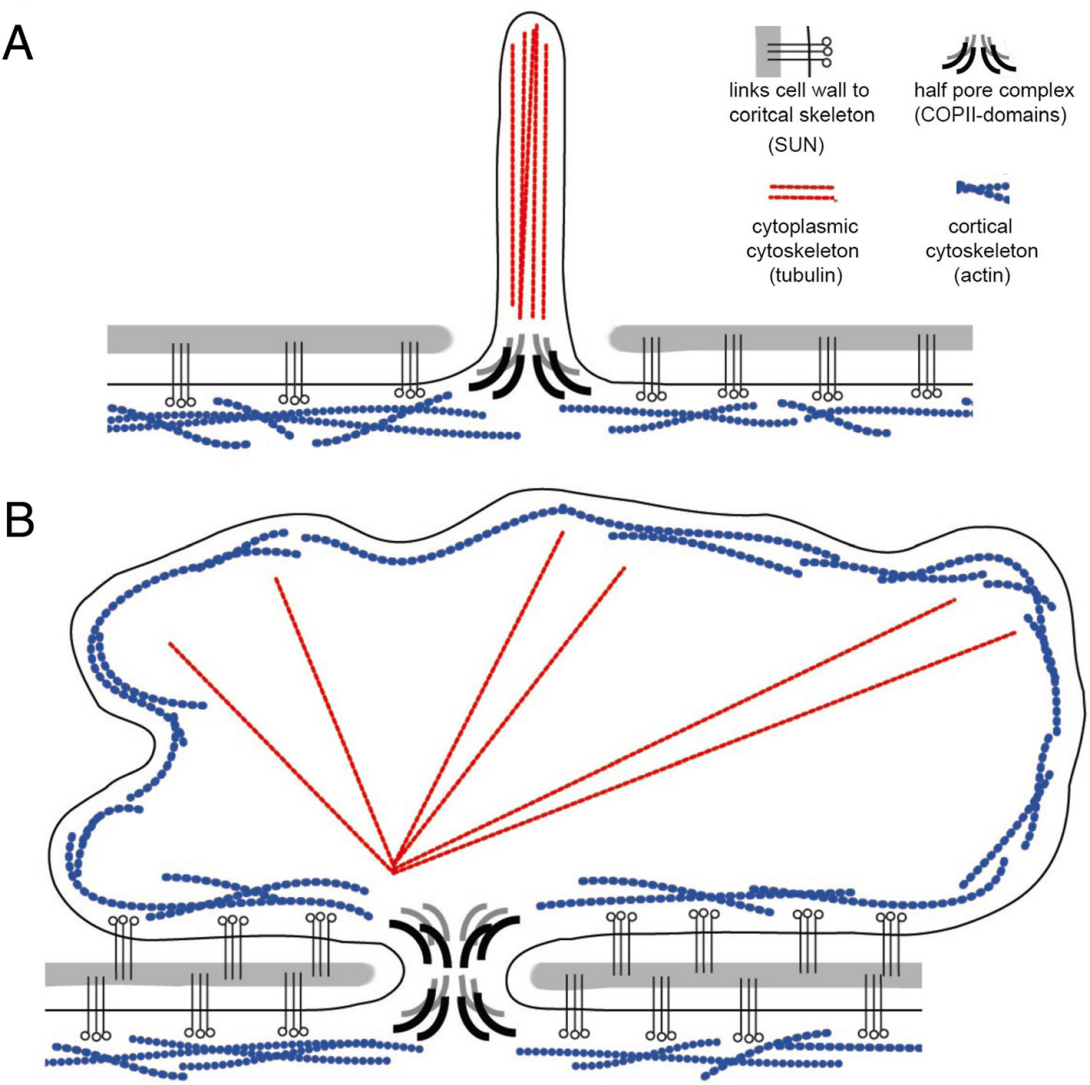
**Model for the evolution of nuclear pores and cytoplasmic blebs. (A)** Membrane protrusions are formed that extend through holes in the cell wall (S-layer, shown in gray) of the eukaryote ancestor. Protrusions could initially have been coated with an S-layer that was later lost. We propose that protrusions gained structural support at their bases from proteins with seven-blade β-propeller domains (homologs of nucleoporins and COPII coatomers), which stabilize positively curved membranes. Additionally, blebs may have been stabilized by an internal cytoskeleton (red), like that provided by microtubules in modern day flagella, and by components of LINC complexes that connect the cell membrane (and underlying structures) to the S-layer (gray). **(B)** Lateral spreading of the bleb is aided by the movement of LINC proteins to the inner bleb membrane and by the recruitment of a second, outer ring of nuclear pore proteins to stabilize positive curvature outside of the cell wall.

Under the inside-out model, the structural components of the nuclear pore constituted the very first eukaryotic innovation, playing an essential role in ensuring the stable attachment of extracellular protrusions to the cell body. This hypothesis leads one to expect the outer ring of the NPC to be the most highly conserved portion of the complex - as is the case [72]. Moreover, in line with the idea that the complex evolved to stabilize long-lived protrusions, NPCs are among the most stable proteins in eukaryotic cells [75,76].

Within eukaryotes, there is now abundant evidence that structural components of the nuclear pore (for example, Nup107) are homologous to COPII proteins that drive the budding of endomembrane vesicles [40,68,69,72,74,77,78]. They even share subunits in common (Sec13/31) [79]. This led Devos and collaborators to propose the protocoatomer hypothesis [40,80], which assumes an outside-in origin of the nucleus. They proposed that an ancestral protein involved in maintaining positive curvature around vesicles and at the edges of ER sheets underwent gene duplication, and some copies became specialized to function at nuclear pores - which are seen as being topologically equivalent to the edges of ER sheets.

Under the inside-out model, this same homology is interpreted differently: proteins whose original function was to stabilize positive membrane curvature in the nuclear pore were later co-opted for a new function in vesicle formation. To distinguish between these theories it will be important in future work to conduct a phylogenetic analysis of COPII and NPC proteins, rooted with appropriate prokaryotic sequences, to determine if the trees better support the inside-out or protocoatomer interpretation.

### Selective pressures for protrusion growth: an increasingly intimate association with symbiotic proto-mitochondria

We suggest that external protrusions evolved in the original proto-eukaryote to facilitate resource exchange with ectosymbiotic bacteria that ultimately gave rise to modern day mitochondria. The presence of a significant α-proteobacterial contribution to all eukaryotic genomes, even those that lack mitochondria or hydrogenosomes, shows that a close association with mitochondria evolved in the eukaryotic stem lineage [7,8,19,30,42].

A number of modern bacteria form ectosymbiotic associations with specific hosts (for examples, see [81-83]), including archaeal species. A good example of this, illustrated in Figure 2, is the archaeon Candidatus *Giganthauma karukerense*, whose cells appear coated with epibiotic γ-proteobacteria [84]. This illustrates the ecological plausibility of progenitors of mitochondria being ectosymbiotic bacteria that entered into a metabolic mutualism with the progenitor of the host cell. This type of association would be augmented by a progressive increase in host cell surface area. Something similar is seen in the foraminiferan *Bolivina pacifica*, which increases its membrane surface area in parts of the cell that underlie prokaryotic ectosymbionts [85]. Thus, selection for an increase in the surface area available for metabolic exchange with ectosymbiotic bacteria could have driven production and proliferation of extracellular protrusions.

The nature of the material exchange between the eukaryotic host and proto-mitochondria has been a matter of debate [23,24,44,45]. Possibilities include hydrogen, sulfur, hydrogen sulfide, organic acids, and ATP. It is worth noting that the inside-out model is consistent with the biochemical and ecological ‘hydrogen hypothesis’ [45,86], except that, by identifying eocytes as the most likely host, a methanogenic host metabolism seems unlikely. Nonetheless, the idea that efficient transfer between proto-mitochondria and a symbiotic archaeon selected for an increasing surface area of contact is shared by both the hydrogen and inside-out hypotheses.

Current data suggest that mitochondria are most closely related to the α-proteobacteria [33]. Typically, analyses have identified mitochondria as very close relatives of Rickettsiales [87,88], a group of intracellular parasites of eukaryotes that co-opt the host cell’s phagocytic machinery to enter cells in food vacuoles, and then enter the cytoplasm proper by lysing the food vacuole membrane [87]. The fact that Rickettsiales live inside eukaryotic cells and have, therefore, had many opportunities to experience gene exchange with mitochondria, combined with the fact that mitochondria and Rickettsiales have very low GC content compared to other α-proteobacteria, means that the apparent close relationships of these two groups could be artifactual [33]. However, even if mitochondria are eventually confirmed as close relatives of Rickettsiales, for reasons discussed below we do not consider it likely that the ancestor of mitochondria entered its proto-eukaryotic host by phagocytosis. Instead, we propose that mitochondria are derived from ectosymbionts, and that the endoparasitic capabilities of Rickettsiales evolved later.

Material exchange with a mutualistic epibiotic bacterial community would have favored both loss of the S-layer overlying protrusions and lateral expansion of protrusions into larger blebs, increasing both cell volume and surface area (Figure 1B-D). Such an expansion would have trapped populations of bacteria between the folds of adjacent blebs and the underlying cell wall (Figure 1C,D). This would have ensured sustained close contacts between host cytoplasm and proto-mitochondria, increasing the probability of vertical proto-mitochondrial inheritance, and helping to exclude parasitic microbes.

At some point, either before or after further elaboration of the cytoplasmic compartment (Figure 1E,F), mitochondria moved into the cytoplasm by penetrating the ER membrane. This seems plausible since rickettsialean bacteria, which are often found within the ER and Golgi of modern eukaryotes [89], gain entry to the cytoplasm proper by lysis of the confining host-cell membrane [87]. It is striking in this light that mitochondria in modern eukaryotes retain close metabolic, physical, and regulatory linkages with ER [90]. The ER has even been found to play a critical role in mitochondrial fission [91,92].

### The expansion of extracellular protrusions and the generation of an incipient endoplasmic reticulum and perinuclear space

The extent to which membrane protrusions swelled beyond the S-layer would have depended on the relative osmotic pressure of the cell and its environment, and the sophistication of osmoregulation. While data on osmoregulation in Archaea remain sparse [93,94], it is noteworthy that many archaeal cells live in conditions of high external osmolytes where the thinning or loss of the S-layer would not cause cells to burst. *Thermoplasma*, for example, appears to lack a cell wall entirely [95].

We propose that with the progressive growth of the external (cytoplasmic) compartment, adjacent blebs pressed against one another to generate a continuous network of inter-bleb crypts, homologous to the lumen of the nuclear envelope and the ER of modern eukaryotes (Figure 1D). This would provide a simple explanation for the continuity of ER and the nuclear envelope, a common feature of all eukaryotes [96] (even within the context of syncytia generated via incomplete cell division [97,98]). Furthermore, since the location of the original glycoprotein-rich archaeal cell wall is topologically equivalent to the perinuclear space in modern eukaryotes, the model parsimoniously explains why the N-linked glycosylation pathway, which operates in the lumen of the nuclear envelope and ER to modify proteins destined for secretion, is homologous to that used to modify S-layer proteins in Archaea [99,100].

The stabilization of blebs would have been facilitated by the evolution of an outer ring of nucleoporins supporting a second area of positive curvature on the outside of the cell wall, giving rise to the partial inside-out symmetry of the NPC (Figure 3). Additionally, the nucleus would have been stabilized by the co-option of proteins used to anchor the cell membrane to the inner surface of the S-layer. Under the model, these would have given rise to LINC complexes [101,102]. In vertebrates, where nuclear envelope structure is best understood, the key components of LINC complexes are SUN-domain proteins on the nucleoplasmic side and KASH-domain proteins on the cytoplasmic side [101-103]. Torsin, which sits within the perinuclear space, interacts with SUN-KASH domain proteins [102,104], as well as other linkers [105,106]. These proteins function together to ensure the structural integrity of the nuclear envelope. Moreover, Torsin has been shown to play a role in nuclear bleb formation during ribonuclear protein granule export [107,108] and in the control of ER morphology [109]. Some of these functions are clearly ancient, given that SUN-domain proteins play a similar role in plant nuclei [110,111].

Under the inside-out model, it seems likely that LINC complexes would be descended from archaeal S-layer glycoproteins. It is therefore noteworthy that many perinuclear components of LINC complexes are N-glycosylated. We speculate further that LINCs originally functioned to connect the archaeal plasma membrane (and perhaps cytoskeleton) to the S-layer. Later, following the growth of cytoplasmic blebs, it is easy to imagine how gene duplication and the recruitment of new proteins could have connected the inner membranes of each bleb to remnants of the S-layer to create a perinuclear lumen and a double nuclear envelope. Although this scenario is attractive, most of the what we know about the structure of the nuclear envelope comes from animal systems, and the identity of potential homologs in archaea remains unknown. Torsin, for example, is a member of an animal-specific subfamily of AAA + ATPases [112], and so may not be a good candidate for an ancestral S-layer protein co-opted to help generate a nuclear envelope. By contrast, SUN-domain protein are found in all eukaryotic groups and have structural homology to carbohydrate-binding motifs [72], which are also present in some archaeal proteins. Thus, it will be important to characterize the closest archaeal homologs of these nuclear envelope scaffolding proteins to determine whether they play a role in anchoring the plasma membrane to the S-layer, as we predict.

### A switch in lipid metabolism

The majority of the structural lipids within eukaryotic cell membranes are quite distinct from archaeal lipids [113,114]. In fact, they bear many similarities to those found in bacteria [115]. Bacterial and eukaryotic membranes are primarily composed of ester-linked, straight-chain fatty acids and utilize glycerol-3-phosphate lipids, whereas archaea have ether-linked fatty acids derived from highly methyl-branched isoprenoids and utilize a glycerol-1-phosphate backbone [114]. Additionally, both eukaryotes and some bacteria, but not archaea [116], produce triterpenoids (for example, hopanoids and sterols) that help modulate membrane fluidity. It is noteworthy, therefore, that a significant fraction of eukaryotic genes assigned a function in lipid metabolism and transport have their closest prokaryotic relatives in α-proteobacteria [33] - including genes involved in eukaryotic sterol synthesis [116,117]. This strongly suggests that eukaryotes acquired their bacterium-like lipids from mitochondria. This conclusion is reinforced under the eocyte hypothesis, which embeds the eukaryotes within the Archaea, implying a late and dramatic switch from archaeal to bacterial lipid biochemistry.

It seems likely that the transfer of genes for lipid biosynthesis from proto-mitochondria to proto-eukaryotes occurred prior to the development of an elaborate vesicle trafficking system and phagocytosis. If this were not the case, one would have to postulate that numerous proteins that had evolved to manipulate archaeal membranes tolerated the shift towards bacterial membranes, which have distinct chemical and biophysical properties [113,118]. While one can envisage a few membrane-interacting proteins, especially those with simple modes of interaction (as seems to be the case for ESCRTIII [119]), being able to retain functionality during a transition from archaeal to bacterial membranes, we think it likely that most membrane-manipulating machinery of eukaryotes arose *after* membranes were bacterium-like. Furthermore, it is hard to see how processes like phagocytosis, which rely both on a large cell size and dramatic, energy-intensive membrane remodeling events could have occurred in an archaeal proto-eukaryote lacking mitochondria [1].

The contention that phagocytosis evolved after the acquisition of mitochondria (as previously suggested [8]) can be further justified by consideration of the physical properties of archaeal lipids. Archaeal membranes typically retain their physical properties across a wide range of temperatures, whereas bacterial and eukaryotic membranes are tuned to keep them close to the phase transition boundary at physiological temperatures [118]. The latter property is thought to allow the formation and dissolution of distinct lipid domains, which permits the dynamic and reversible membrane deformations that are characteristic of eukaryotic cells [120]. These considerations support the idea that the physico-chemical properties of bacterial membranes were an essential precursor to the evolution of dynamic mechanisms such as endocytosis and phagocytosis.

These facts are hard to reconcile with outside-in models, which typically view phagocytosis as the means by which proto-eukaryotes established a close, symbiotic relationship with proto-mitochondria. By contrast, the inside-out model implies that symbiosis arose by the passive trapping of proto-mitochondria in inter-bleb spaces, and did not require complex membrane manipulating machinery besides the ability to generate protrusions - a feature common in many modern-day archaea.

Under the inside-out model, the structural lipids present in modern eukaryotes would have been first acquired from mitochondria via traffic across ER-mitochondrial contact sites, which are conserved across eukaryotes and apparently ancient [121]. Given this, there are a number of striking observations. First, mitochondria retain a critical role in eukaryotic fatty acid metabolism and in lipid synthesis, generating many of their own lipids, such as cardiolipin [113,122]. Second, the ER is the major site of lipid and membrane synthesis in modern eukaryotes, with many of the enzymes involved found concentrated at ER-mitochondrial contact sites [123]. And third, connections between ER and mitochondria remain important sites of lipid traffic in modern eukaryotes [124-126]. Thus, the spatial organization of lipids and lipid synthesis in modern cells is easy to understand under the inside-out model as a by-product of the gradual evolution of a symbiotic relationship between the host and mitochondria (the original site of endomembrane lipid synthesis) situated in the spaces between cytoplasmic blebs.

For a time it is likely that membranes were formed that contained a mixture of archaeal and bacterial lipids [127] prior to gradual reductions in the archaeal contribution. The primary use of only one type of structural lipid may have been driven in part by the difficulties of reconciling metabolic pathways that use different chiral forms of the lipid glycerol backbone, with the mesophilic environment removing any intrinsic benefit of ether-linked lipids. Interestingly, though, modern eukaryotic cells do produce some lipids with ether-linkages [128,129], some of which have been implicated in the generation of mechanically rigid membranes during cell division [130]. These facts raise the possibility that use of archaeon-like lipids in cell division helped ESCRTIII to survive the transition from archaeal to eukaryotic cell biology.

In contrast to the structural lipids of eukaryotes, inositol lipids, which are ubiquitous in eukaryotes but represent a tiny fraction of total lipids in membranes [125], are common to eukaryotes and archaea, but not bacteria [131]. This implies that inositol metabolism was originally associated with the proto-nuclear compartment, thus explaining why inositol lipids are actively imported into mitochondria rather than being synthesized there [126,132]. This may also account for the fact that inositol lipids, and the enzymes that generate them, are found in the nuclei of modern eukaryotes - something that has long perplexed researchers in the field [133,134]. Instead of a structural role, inositol lipids are important regulatory molecules, modulating cell growth [135,136] and marking cytoplasmic compartment identity [137]. This is reasonable under the inside-out model: inositol derivatives were present throughout eukaryotic evolution, allowing their phosphorylation states to be deployed as signals [135,136] for facilitating nuclear control over an increasingly large and elaborate cytoplasmic compartment.

### The mitotic cycle and cell division in the proto-eukaryote

Despite the presence of blebs and proto-mitochondria at early stages in its evolution (Figure 1A-D), the proto-eukaryote would have had the same topology as the ancestral eocyte. It retained a single, continuous bounding membrane, albeit one that was much more extensive and contorted than the ancestors’. Thus, at this stage there would have been no distinction between nuclear division and cell division. Moreover, cell cycle progression and cell division would have likely been regulated in a manner similar to that seen in modern day Archaea, and using homologous proteins [58]. Likewise, proteins controlling chromosomal architecture (histones) and DNA replication are of archaeal origin [138].

**Figure 4:**
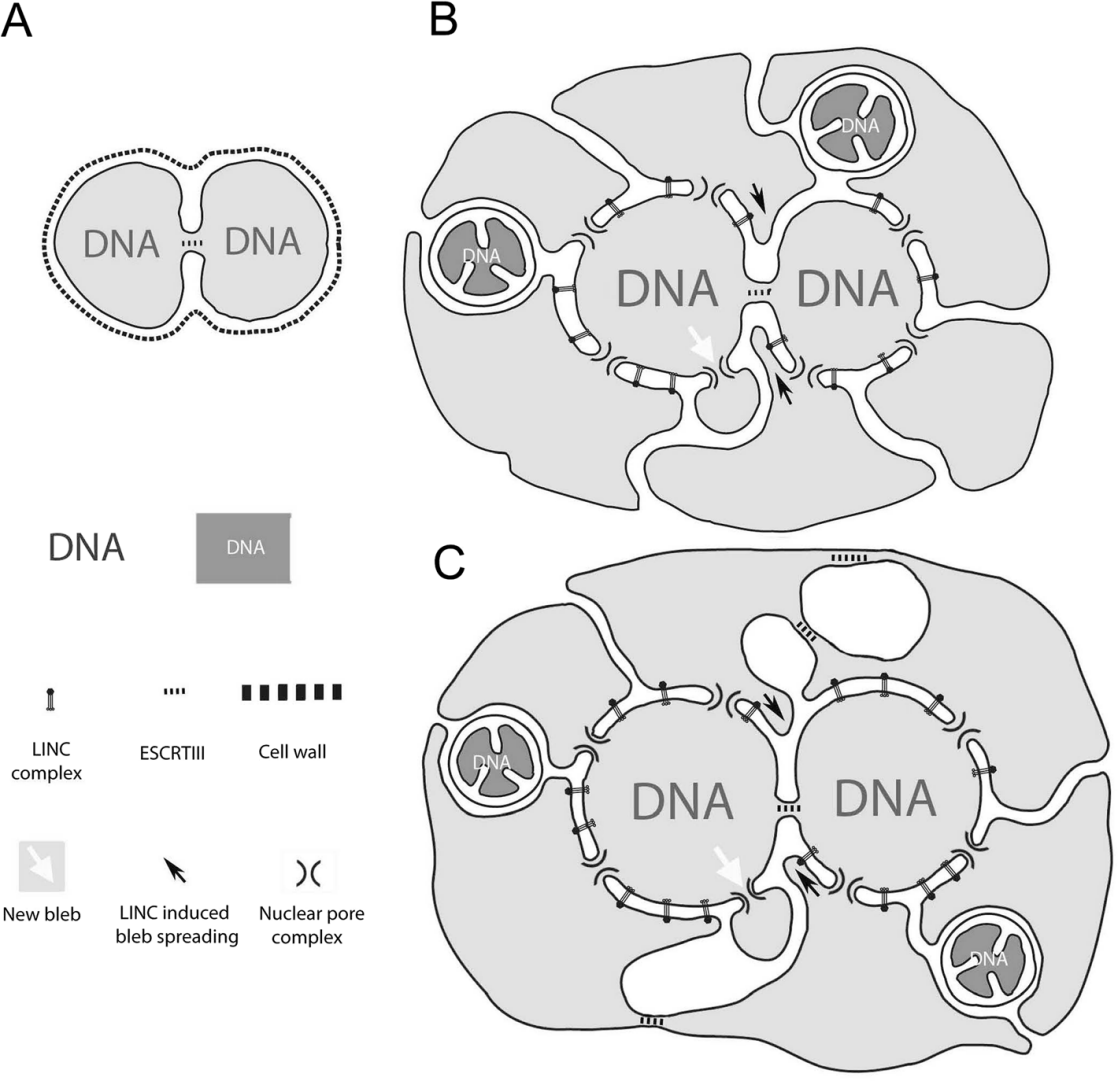
**Model for the evolution of cell division.** Cell division is depicted for the ancestral eocyte **(A)**, and at two intermediate stages in the evolution of eukaryotes, before **(B)** or after **(C)** bleb fusion. Following the acquisition of blebs, ESCRTIII is used to drive the scission of cytoplasmic bridges connecting cells (likely aided by the archaeal-derived actin cytoskeleton [51])*,* while LINC complexes and the formation of new nuclear pores restore cell and nuclear organization following division. Mitochondrial segregation is likely aided by host induced Dynamin-mediated scission within the endoplasmic reticulum (not depicted), as observed in modern eukaryotes [91].

Strikingly, in many archaea, the scission event completing cell division is driven by the action of the ESCRTIII complex [56-58], just as appears to be the case in eukaryotes [139]. Under the inside-out model, it is relatively easy to see how cell division could have been achieved in an early proto-eukaryotic cell, even one that had links between blebs, using pre-existing ESCRTIII machinery (Figure 4). After division, each daughter cell would have acquired a subset of the nuclear pore-associated blebs, with naked cell surface being covered by the movement of pores and through the action of LINC complexes [101], which would attach flanking bleb membranes to the exposed portion of the proto-nucleus (Figure 4). However, in a proto-eukaryote with a well-developed cytoplasmic compartment, the simple division of the nuclear compartment would not have guaranteed a fair segregation of cell mass between the two daughter cells. After loss of the original cell wall, this problem could have been solved through the evolution of partially open mitosis (Figure 4). Because the inner nuclear membrane is topologically continuous with the outer bleb membrane, this would have required little additional innovation, only the partial disassembly of nuclear pores and LINC complexes as seen in some eukaryotic cell divisions [140]. Following division, the nuclear-cytoplasmic boundary would have been re-established through the rebinding of nuclear membranes by chromosome-associated NPC and LINC components.

From this type of mitosis, it is easy to see how further loss or remodeling of LINC complexes could lead to a more complete loss of the nuclear envelope associated with fully ‘open’ mitosis. In this light, it is important to note that although the initiation of an open mitosis is often referred to as being triggered by nuclear envelope ‘breakdown,’ this is a misnomer, because in the majority of eukaryotes there is no breakdown of the membrane. Instead, there is a loss of compartment identity as nuclear and cytoplasmic compartments mix and nuclear membranes become indistinguishable from cytoplasmic ER [141-144]. Under the inside-out model it is easy to see that open and closed mitosis are not as different as often assumed, and to imagine cells switching between open and closed modes of mitosis by modifying the extent to which LINC and NPCs remain associated with the nuclear membranes during cell division. This offers an explanation for the frequent occurrence of evolutionary transitions between these two modes of mitosis [2,145].

### The differentiation of nuclear and cytoplasmic compartments

Under the inside-out model, the recruitment of additional proteins to the NPC enabled the controlled movement of membrane lipids and the flow of aqueous material between the nuclear and bleb (cytoplasmic) compartments. This includes the regulated transport of mRNA and ribosomes [146,147] to generate distinct domains of protein translation: nuclear and cytoplasmic. In such a situation, it is easy to imagine that it might be beneficial for certain transcripts to be translated in the cytoplasmic domain and that this might have resulted in the evolution of mechanisms for targeting some transcripts for transport to the cytoplasm and for preventing their premature translation in the nucleus. We speculate that mRNA cap formation and polyadenylation evolved originally for this purpose: tagging certain transcripts for translocation through the nuclear pore and limiting intranuclear translation. It is noteworthy that, in some systems, mRNA processing [148,149] and mRNA export [150] are regulated by phosphoinositol lipids which, as suggested above, might have had an ancestral role in coordinating growth of the nuclear and cytoplasmic compartments.

Through the regulated transport of mRNA and proteins between nuclear and cytoplasmic compartments it would have become possible to separate core metabolic processes in the cytoplasm from DNA replication, transcription, and ribosome assembly in the nucleoplasm. This would have limited the exposure of the genome to the dangerous by-products of metabolism (for example, reactive oxygen species generated in mitochondria). In addition, the separation of transcription, RNA processing, and translation provided more control over gene expression, making possible, for example, the evolution of alternative splicing [151].

### First steps in the evolution of eukaryotic secretion

In the ancestral eocyte, signal recognition particle (SRP) complexes would have driven the secretion of proteins through the plasma membrane into the environment. From this starting point, we propose that some SRP complexes, which are apparently of archaeal rather than mitochondrial origin [152], became concentrated on bleb membranes close to the original cell body. This caused ribosome-mediated protein export to be directed into the perinuclear lumen and the proximal ER; thereby generating rough ER. This altered plumbing would have limited the exposure of newly secreted proteins to the environment, and would have generated an extracellular pool of highly concentrated protein that could be subjected to complex modifications prior to its diffusion beyond the cell.

While there are few cases of N-linked glycosylation in bacteria, archaea have an N-linked protein glycosylation pathway that has many similarities to that seen in eukaryotes, and which operates to modify secreted and transmembrane proteins [99,153]. The glycosyltransferases that add sugar groups to asparagine residues on secreted archaeal proteins are closely related to the equivalent eukaryotic enzymes [100]. These data support the idea that the machinery governing eukaryotic protein glycosylation was inherited from archaea - where N-glycosylation appears to contribute to the structural integrity of the S-layer [50].

Under the inside-out model, the glycosylation machinery would have been situated in the extracellular space at the base of cytoplasmic blebs early in the evolution of eukaryotes - equivalent to the lumen of the nuclear envelope and ER of modern eukaryotes (see Figure 1). Thus, the model provides a simple explanation for the origin of the machinery governing protein glycosylation, the site of glycosylation with eukaryotic cells, and its function in secretion.

### Establishing cytoplasmic continuity in the proto-eukaryote

The increase in the relative size of the cytoplasmic compartment would have been aided by the evolution of an increasingly sophisticated cytoplasmic cytoskeleton and by the thinning or loss of residual cell wall material (Figure 1C). Moreover, because it would have occurred through the expansion of cytoplasmic blebs, this increase in mass could have been achieved without a large change in surface-to-volume ratio.

At the same time, the growth of individual cytoplasmic compartments would have necessitated the evolution of machinery to generate connections between adjacent blebs in order to integrate processes across these increasing large cells (for example, facilitating accurate cell division and cell polarization). While topologically equivalent endomembrane fusion events have not, to our knowledge, been studied in modern eukaryotes, the presence of fenestrae in Golgi [154] and ER [155] suggests the likely operation of such mechanisms. Moreover, the topological transformation required to link cytoplasmic compartments is identical to the one proposed to function during the insertion of nuclear pores into interphase nuclei (see below). Thus, it is possible that proteins generating ER fenestrae and bleb-to-bleb connections are related to Ndc1, POM121, and Gp120, which are thought to facilitate the fusion of the inner and outer nuclear membranes during nuclear pore insertion [156]. This type of fusion activity is also likely to have been a pre-requisite for the evolution of sex in eukaryotes.

### Formation of the plasma membrane

The final topological innovation under the inside-out model was the formation of a single, continuous plasma membrane. The development of a distinct plasma membrane could have been achieved by large blebs engulfing the rest of the cell (Figure 1F). In this case, a topological transformation would be needed to seal the residual tubular hole connecting the ER inter-bleb space to the external environment. This could be done by assembly of Dynamin around the cytoplasmic portion of the tube [157], which would lead to scission of the tube and the formation of a topologically distinct plasma membrane and an internal ER network sealed off from the environment. Dynamin would have been present, likely having been acquired from mitochondria [158]. Alternatively, peripheral regions of the ER might have accumulated fusogenic proteins, similar to those involved in gamete fusion, which promoted promiscuous fusion of distal bleb membranes. Again, residual, tubular connections between the ER and plasma membrane could have been severed by Dynamin.

Following the generation of a topologically distinct plasma membrane, we think it likely that dividing cells needed a way to generate the force necessary to bring the plasma membrane into a favorable alignment for subsequent ESCRTIII-mediated scission. This would have likely led to the evolution of a cytokinesis ring, perhaps based upon the pre-existing archaeal actin cytoskeleton [51], which has been implicated in cytokinesis. Thus, while there is certainly scope for much subsequent evolution in the mechanisms used for cytokinesis and karyokinesis, our model predicts that the molecular mechanisms of cell and nuclear division were initially very similar, as suggested by data in fission yeast, where a nuclear fission pathway has been identified and shown to be actin-dependent [159].

Once systems were in place for nuclear and cellular division in the presence of a plasma membrane, a major challenge would be the spatial coordination of nuclear and plasma membrane scission. Apparently, solutions to this problem evolved multiple times, as judging by the diverse modes of cytokinesis seen in modern eukaryotes. For example, budding yeast, whose cells undergo a closed mitosis, solve this problem by positioning the nucleus across the bud neck, a process that makes ESCRTIII dispensable for division under most conditions [160]. By contrast, animals with an open mitosis may use the force generated through the cytokinetic ring to help sever the ER remnants of the nuclear envelope [142,144].

Only after the formation of a continuous plasma membrane would eukaryotes be able to evolve cell walls. This explains why eukaryotes lack S-layers. Furthermore, if the last eukaryotic common ancestor lacked a wall, it is easy to understand why different eukaryotic phyla acquired biochemically distinct cell walls.

The late evolution of the plasma membrane also suggests that there is no reason to expect every nucleus to be contained within its own plasma membrane-delimited compartment. This is illustrated by syncytial eukaryotes, whether generated through nuclear division or cell-cell fusion [15,161-163], which contain nuclei that lack functional interactions with a plasma membrane. Finally, because complete cell division is not needed for nuclei to achieve distinct developmental identities under the inside-out model (insofar as nuclei naturally ‘control’ a set of nuclear pore-associated blebs), it is easy to see how transitions between syncytial and multicellular development can be achieved [162,163]. In this way, the numerous evolutionary transitions between complete and incomplete division, for example in arthropod and plant embryogenesis, are readily explicable.

### Regulated secretion and vesicle trafficking drove elaboration of the cytoplasmic compartment

**Figure 5:**
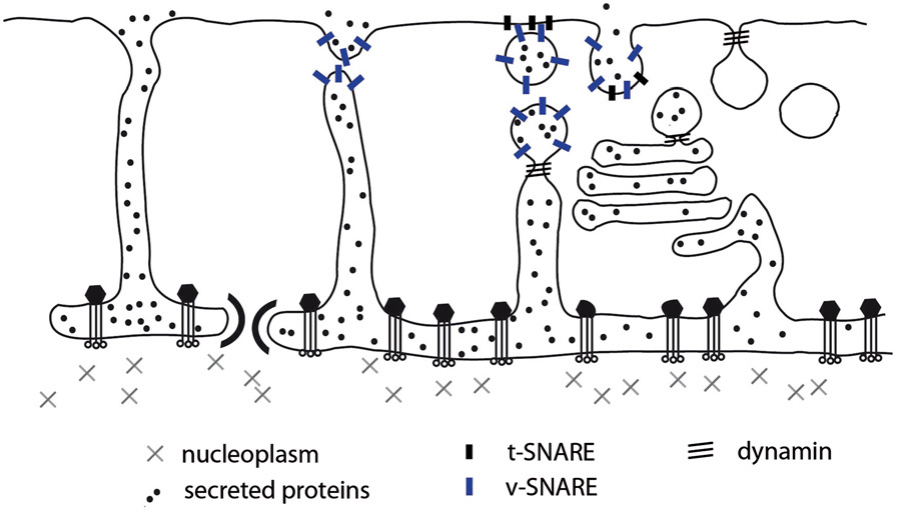
**The stepwise evolution of eukaryotic vesicle trafficking.** From left to right the figure depicts a simple hypothesis for the evolution of the eukaryotic secretion and vesicle trafficking systems. Initially, proteins (black dots) would have been secreted from ribosomes bound to rough endoplasmic reticulum (ER) into the space at the bases of blebs by the Sec translocase and signal recognition particles (SRP) [50]. Secreted proteins could then undergo stepwise processing using machinery adapted from that used to process glycoproteins in the archaeal S-layer (that is, through N-linked glycosylation of asparagine-X-serine or asparagine-X-threonine-containing proteins, and proteolysis [99]). The elaboration of ER tubules and local membrane bending regulated by the Sar1 GTPase, in the presence of generic SNAP Receptors (SNAREs) (blue bars), would have enabled the transient fusion of ER to the outer cell membrane, releasing these glycosylated proteins into the extracellular space. These transient openings would have been closed by Dynamin-mediated fission. Specialized SNARE proteins (differently colored bars) and Dynamin (triple diagonal lines), would then have generated vesicular intermediates to better regulate secretion. The intercalation of additional processing steps and the diversification of these protein families would have yielded compartment-specific paralogs, together with the evolution of regulatory Arf and Rab GTPases, and a Golgi compartment. Finally, membrane bending machinery together with Dynamin, actin, and Rho family GTPases would have been co-opted to drive endocytosis, phagocytosis, and the development of the modern retrograde trafficking pathway.

The presence of a plasma membrane would have given the cell tighter control over material exchange with the environment, preventing direct diffusion into and out of the ER space. For example, it would have ensured the vertical inheritance of mitochondria - facilitating its co-evolution with the host. It is important to note, however, that under the model a distinct plasma membrane could not have been maintained until systems of membrane delivery and removal were already in place. This would have relied upon modifications to pre-existing secretory pathways. A likely scenario (Figure 5) is that transient fusion of ER tubules and ER-derived vesicles with the newly formed outer cell membrane contributed to membrane delivery and removal at early stages following plasma membrane formation. While there is old literature supporting the ER being continuous with the plasma membrane in some eukaryotes [164,165], this remains to be re-confirmed. Later, the intercalation of cargo-modifying steps between the ER and the bounding membrane would have enabled better regulation of the plasma membrane, secretion, and retrograde traffic.

The rooting of the eukaryotic tree remains uncertain, though there is now some support for a root between the Opisthokonta (which includes animals and fungi) and the rest of the eukaryotes [166,167]. Under this rooting, any traits shared by animals, fungi, and plants should be ancestral for eukaryotes as whole. This would seem to include almost all aspects of the endomembrane trafficking system because the last eukaryotic common ancestor had a full complement of molecular machines involved in vesicle trafficking [168]. Nonetheless, some tree topologies suggest that members of Amoebozoa or Excavata *could* retain ancestral characteristics [167]. In light of this it is noteworthy that the excavate *Giardia* has some features that resemble a hypothetical intermediate. Specifically, there is some evidence that secretion in *Giardia* may be accomplished by the direct, transient fusion of ER tubules with the outer cell membrane [169]. Furthermore, secretory vesicles in *Giardia* may be pinched off from the ER and fuse with the plasma membrane without any intervening processing steps [170]. However, because *Giardia*’s cell biology is complex and varies through its life cycle [171], it is not clear if it truly manifests transitional traits.

**Figure 6:**
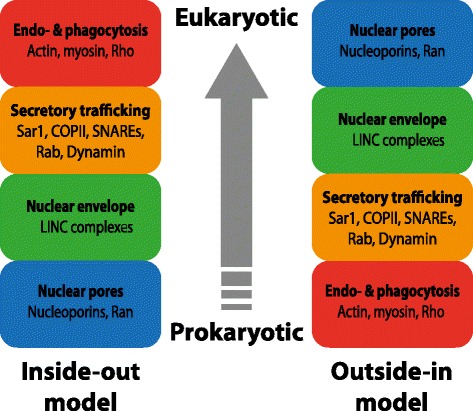
**Comparison of the predicted ordering of cellular innovations, and the corresponding molecular machines, under the inside-out and autogenous outside-in models.** Ran, Rab, Sar1, and Rho refer to small GTPase subfamilies. Abbreviations: LINC = Linker of Nucleoskeleton and Cytoskeleton; COPII = Coat protein II; SNAREs = SNAP Receptors.

Strikingly, the order in which the secretory and endosomal trafficking systems evolved is almost precisely opposite under inside-out and outside-in models (Figure 6). Phylogenetic analysis of the Ras superfamily, which includes proteins functioning in diverse cellular trafficking steps, has the potential to discriminate between these two hypotheses. Under the inside-out scheme, Ran GTPase function would have been the first to evolve, given its role in regulating traffic between cytoplasmic and nuclear compartments (with a possible additional role in nuclear pore assembly and insertion). Subsequent gene duplications would have enabled a copy to acquire Sar1 function, enabling it to promote COPII-mediated vesicle budding from the ER (Figure 5), followed by Arf and Rabs, which are involved in vesicle trafficking. The last addition to the family would have been Rho GTPases, which regulate the actomyosin cortex underlying the plasma membrane, endocytosis, and phagocytosis.

Rooted phylogenetic analyses of the Ras superfamily [172-175] generally support three major clades: Ran, Sar1/Arf, and Rab/Ras/Rho, which can be interpreted as specializing in nuclear transport, secretion, and endocytosis, respectively [172]. Based on phylogenetic inference it has been argued that, as predicted by the inside-out model, Ran and Sar1 functions predate Rab and Rho functions [173]. While other interpretations of the same trees are possible, if one is willing to assume that functions evolving earlier accumulate more critical functions and, thus, show greater evolutionary conservation, it is noteworthy that Ran and Sar1 tend to be present in a single copy per eukaryote genome and are very highly conserved, whereas Rab, Ras, and Rho proteins have very divergent sequences and are present in large and variable numbers across eukaryotes.

### The origin of eukaryotic cilia

The final widespread organelle of modern eukaryotes to consider is the cilium, flagellum, or undulipodium [32]. Cilia are stable plasma membrane-bound protrusions supported by a stable neck complex and an internal cytoskeleton (microtubules) that have obvious similarities to the protrusions that we envisage as the starting point for the evolution of the cytoplasm (Figure 2A). Cilia can yield extracellular vesicles [176,177] like those generated when archaeal protrusions are pinched off. Furthermore, just like proto-cytoplasmic protrusions, some cilia have become elaborated during evolution to yield larger and more complex structures, like photoreceptors [178].

While not a central feature of the model, the inside-out theory suggests that the machinery for generating cilia might have similarities to that generating protrusions early in the evolution of eukaryotes. In this light, it is striking that Ran GTPase works together with importins to regulate traffic both across nuclear pores and into cilia [179-181] and that both structures have a similar size exclusion limit [182]. Indeed, some have suggested nucleoporin-like proteins may be situated at the base of cilia [182], although current data cast doubt on this claim [183]. Nevertheless, under the inside-out model one can readily imagine common machinery that arose early in the evolutionary path of eukaryotes being co-opted later to give rise to cilia on the new eukaryotic plasma membrane.

### Additional predictions of the inside-out model

In previous sections we described the inside-out theory and its consistency with diverse aspects of eukaryotic cell biology. In addition, Table 2 summarizes a number of predictions of the inside-out model relative to outside-in models.

**Table 2:** **Predictions to better differentiate inside-out and outside-in models**

**Prediction under the inside-out model**	**Prediction under the autogenous outside-in model**	**Prediction under the endosymbiotic outside-in model**
Two prokaryotic genomes (nuclear and mitochondrial) contributed to the ancestral eukaryotic genome.	Two prokaryotic genomes (nuclear and mitochondrial) contributed to the ancestral eukaryotic genome.	Three prokaryotic genomes (nuclear, cytoplasmic, and mitochondrial) contributed to the ancestral eukaryotic genome.
Mitochondria or mitochondrially derived genes are present in all eukaryotes.	Eukaryotes might be found that have no evidence of having mitochondria in their ancestry.	Eukaryotes might be found that have no evidence of having mitochondria in their ancestry.
The perinuclear space contains machinery (for example, N-linked glycosylation) similar to that used to modify the archaeal cell wall.	Homologs of archaeal cell wall proteins, if present, are found on the surface of eukaryotes rather than in the perinuclear space.	The perinuclear space contains machinery related to host food vacuole functions or the cell wall of the endosymbiont lineage.
LINC proteins are homologous to proteins anchoring the archaeal plasma membrane and/or cortical cytoskeleton to the cell wall. Homologs of glycosylated SUN proteins are present in archaeal cell walls.	LINC proteins are likely derived from proteins that function in controlling the shape of membrane-bound vesicles or ER cisternae.	Inner LINC proteins are derived from the periphery of the endosymbiont lineage, outer LINC proteins from the food vacuole of the host lineage.
Homologs of structural nucleoporins are localized to the plasma membrane of eocytes and play a role in stabilizing extracellular projections.	Homologs of structural nucleoporins play a role in invagination of the archaeal plasma membrane.	Homologs of structural nucleoporins play a role in regulated transport into and out of food vacuoles in the host and/or form secretion systems in the endosymbionts.
Nucleoporins form a paraphyletic grade from which COPII-like proteins evolved.	Nucleoporins are embedded in a paraphyletic grade composed of COPII-like proteins that are involved in endosomal trafficking.	Nucleoporins form a paraphyletic grade from which COPII-like proteins evolved.
Synthesis of eukaryotic phospholipids and sterols is accomplished by genes of α-proteobacterial ancestry.	Synthesis of eukaryotic phospholipids and sterols is accomplished by genes of eukaryotic ancestry or by archaeal genes plus genes acquired laterally from bacteria other than mitochondria.	Synthesis of eukaryotic phospholipids and sterols is accomplished by genes of host and endosymbiont ancestry, but not mitochondria.
New interphase nuclear pores are inserted from inside the nucleus at the neck of outward projections from the inner surface of the nuclear membrane.	New interphase nuclear pores are inserted from both inside and outside the nucleus and induce the fusion of inner and outer nuclear membranes to generate a pore.	New nuclear pores arise either from the outside, by host-derived proteins, or from the inside by endosymbiont-derived proteins. No prediction is made as to how they puncture inner and outer membranes.
ER is largely continuous, even in syncytia generated by the suppression of cell division.	ER is largely discontinuous in the absence of ER fusion machinery.	ER is continuous by virtue of deriving from and connecting to the nuclear envelope.
Cytoplasmic continuity must be actively maintained. The cytoplasm associated with individual nuclear pores will show signs of limited connectivity when rates of cytoplasmic fusion are low.	Cytoplasm tends to be continuous.	Cytoplasm tends to be continuous.
Nuclei can, in general, retain distinct domains of action in the context of a syncytium.	Nuclei in syncytia exert local control of adjacent cytoplasm only through recently evolved specialized mechanisms.	Nuclei in syncytia exert local control of adjacent cytoplasm only through recently evolved specialized mechanisms.
Protein functions related to anterograde secretion will tend to be ancestral to functions related to endocytosis, phagocytosis, and retrograde transport.	Proteins functions related to retrograde vesicle trafficking and endocytosis will tend to be ancestral to functions related to anterograde transport and secretion.	Proteins functions related to phagocytosis will tend to be ancestral to functions related to anterograde transport and secretion.
Eukaryotes might be found that retain the ancestral condition of transient connections between ER and the cell’s exterior, or in which there is anterograde but not retrograde vesicle transport.	Eukaryotes might be found that retain the ancestral condition of having a nuclear envelope that is not fully assembled, or that lacks nuclear pores, or in which there is endocytosis but no exocytosis, or in which there in retrograde but not anterograde vesicle transport.	No intermediates will be found.
Transitions between open and closed mitosis are easy and accomplished by changing the stability of LINC complexes and the extent to which nuclear membranes are released into bulk ER by nuclear pore disassembly.	Transitions from open mitosis (the ancestral state) to closed mitosis are difficult to achieve and require the *de novo* evolution of machinery for nuclear fission.	Transitions from closed mitosis (the ancestral state) to open mitosis are very difficult and require rupture and reassembly of the endosymbiont plasma membrane and host food vacuole membrane.
Closed mitosis will utilize ESCRTIII in a manner similar to archaeal cell division.	Closed mitosis will utilize eukaryote-specific molecular mechanisms.	Closed mitosis will utilize molecular mechanisms acquired from the endosymbiont.
The segregation of ER at cell division in a closed mitosis is primarily accomplished by the segregation of nuclear-pore associated cytoplasmic domains.	The segregation of ER at cell division in a closed mitosis is tightly regulated with scission events actively separating domains of ER.	The segregation of ER at cell division in a closed mitosis is tightly regulated with scission events actively separating domains of ER.
Cell cycle control will be dominated by nuclear events, with secondary controls acting to coordinate nuclear and cytoplasmic events.	Cell cycle control may be dominated by cytoplasmic events, with secondary controls acting to coordinate nuclear and cytoplasmic events.	There may be entirely separate mechanisms governing cell cycle control within the nucleus and cytoplasm.
Flagella may utilize proteins homologous to those involved in nuclear pore formation and trafficking.	Flagella formation need not involve proteins like those involved in nuclear pore formation.	Flagella formation need not involve proteins like those involved in nuclear pore formation.

ER, endoplasmic reticulum.

For this comparison predictions of the autogenous outside-in model are guided by previous work [2,32]. Since these predictions mainly relate to the origin of the nucleus and endomembrane system, they are agnostic as to whether mitochondria arose before or after the nucleus [8]. Because of the diversity of endosymbiotic outside-in models, we have tried to summarize the predictions of a generic model in which a single host cell (cytoplasm) engulfed a single endosymbiont (nucleus). Most of the predictions of the inside-out theory are, we think, self-explanatory. However, a few will benefit from a more detailed exposition.

### A new path of interphase nuclear pore insertion

**Figure 7:**
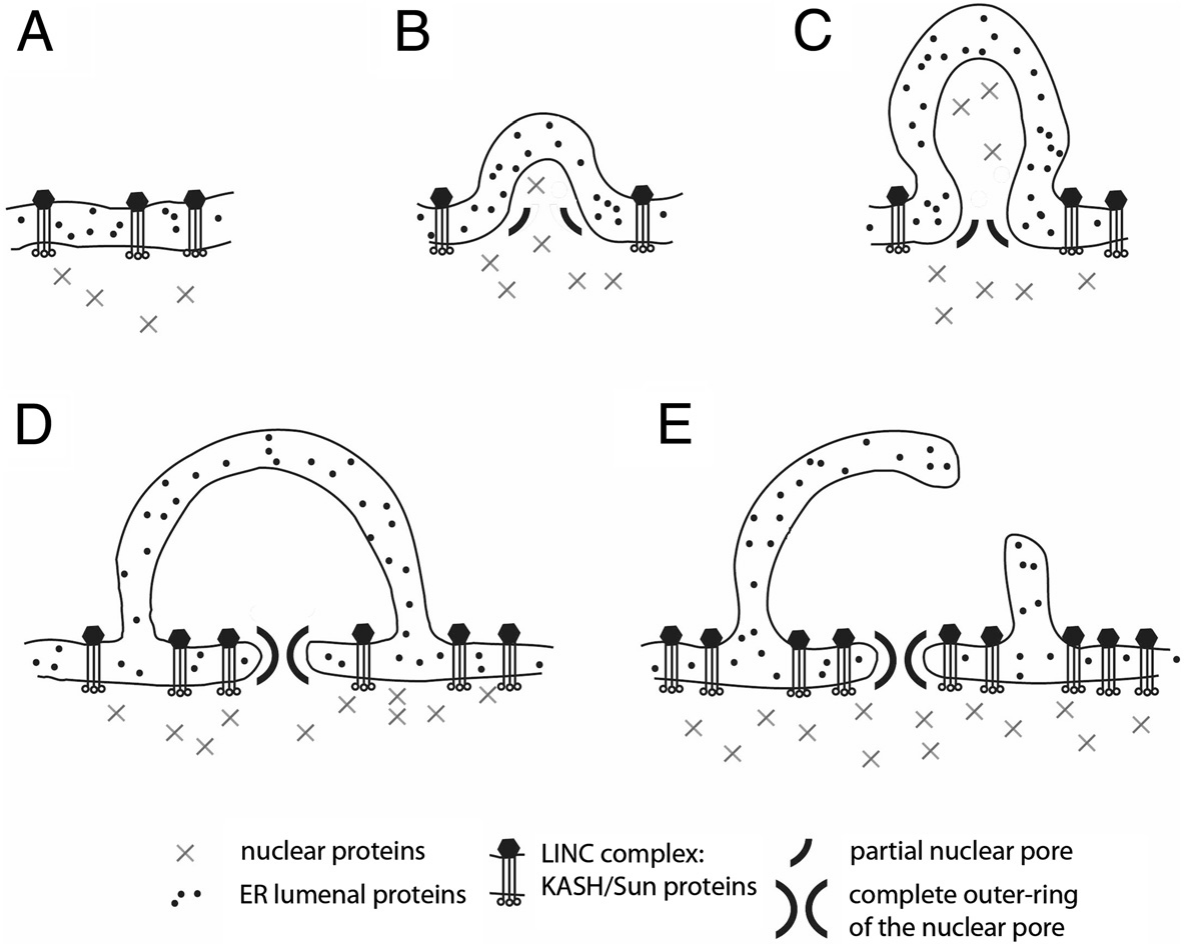
**Predicted mechanism of interphase eukaryotic nuclear pore insertion predicted by the inside-out model. (A)** The nuclear envelope is held together through LINC complexes. **(B, C)** Folds in the inner membrane of the envelope recruit the outer ring of the nuclear pore, composed of proteins with COPII-like domains, to generate a small extranuclear bleb, which is stabilized via the assembly of the complete nuclear pore complex. **(D)** The nuclear pore complex, together with LINC complexes, generates a tight membrane fold at the bud neck. **(E)** The nascent bleb is connected to the rest of the cytoplasm by active bleb-bleb fusion, ensuring cytoplasmic continuity. Note that in this model the continuity of the perinuclear space and the endoplasmic reticulum (ER) is a simple consequence of the mechanism of bleb generation. The relative rates at which bleb expansion **(A**-**D)** and the fusion of cytoplasmic compartments **(E)** occur will determine the size of individual cytoplasmic blebs and the extent of cytoplasmic compartmentalization. Thus, if the compartment fusion reaction **(D**, **E)** is induced immediately, no enlarged blebs would be seen.

The inside-out models yields an explicit prediction as to how new nuclear pores are likely inserted into the envelopes of interphase nuclei. Specifically, using the model as a guide, we hypothesize that the first step in the generation of a new nuclear pore is recruitment of elements of the outer ring of the NPC to bend the inner nuclear membrane outwards (Figure 7), similar to the process generating extracellular protrusions in the original proto-eukaryote. This model contrasts with current models, which typically propose that the inner and outer membranes are bent towards one another to induce fusion prior to nuclear pore assembly and insertion [184].

While definitive data on the details of interphase nuclear pore insertion are lacking, there is some circumstantial evidence that supports our hypothesis. If, as suggested by current models, inserting a new nuclear pore requires membrane fusion before NPC assembly, then the nuclear membrane proteins that regulate such fusion should be essential and conserved throughout eukaryotes. This does not appear to be the case. Transmembrane pore-associated proteins like POM121, which appear to have this fusogenic activity in mammalian cells [156,185,186], show low evolutionary conservation [72] and are absent from trypanosomes [78]. Further, even the most conserved transmembrane pore-associated proteins, Ndc1 and Gp210, which are present in nearly all eukaryotes (but not trypanosomes), lack an essential function in NPC formation [187-190]. The evolutionary lability of these transmembrane NPC proteins contrasts with the outer ring of the nuclear pore, whose components are very well conserved [72].

In our model, proteins that fuse the inner nuclear membrane bleb with the overlying outer nuclear membrane would be recruited sometime after the onset of NPC assembly. As a result, we predict that in cells with a low rate of cytoplasmic compartment fusion it should be possible to observe outward facing nuclear blebs projecting into the cytoplasm with nuclear pores at their bases. Structures with this configuration have been seen in a large number of studies in diverse cell types [191-195]. Moreover, recent studies have reported a function for nuclear blebs in ribonuclear protein granule export [107,108]. We further predict that cells with long-lived nuclear blebs will tend to be those characterized by low rates of compartment fusion, as might be indicated by their having ER and Golgi that lack fenestrae (see below).

### A new biochemical activity that generates links between endoplasmic reticulum-bound cytoplasmic compartments

Under the inside-out model, the eukaryotic cytoplasm is built up of pore-associated blebs, separated from each other by ER. Such a topology might be visible in some modern eukaryotes. However, one would also predict that there would have been selective pressure during eukaryotic evolution for an activity that could induce the formation of connections between adjacent blebs - creating channels across ER. Under the model, protein machines with this activity would have been essential for the generation of a well-integrated cytoplasmic domain. High fusion activity would render the cytoplasm functionally continuous. However, in cells with a slow rate of ER channel formation, one would expect to observe local cytoplasmic discontinuities that would result in the anomalous diffusion of cytoplasmic proteins. Anomalous diffusion is a common feature of eukaryotic cells [196,197], where it has been attributed both to crowding and to physical diffusion barriers [198]. Thus, determining whether cytoplasmic discontinuities and anomalous diffusion correspond to ER-bounded domains provides a good way of evaluating this aspect of the inside-out model.

In addition to predicting patterns of diffusion within single cells, the inside-out model has implications for syncytia generated by nuclear division. Within such syncytia, cytoplasm tends to be under the local control of individual nuclei. As an example of this, individual nuclei in the *Drosophila* syncytial blastoderm establish distinct transcriptional profiles and local compartmentalization [162,199], as do nuclei in syncytial fungi [200-202] and plants [203]. Under the inside-out model it is easy to see how nuclei and associated organelles (for example, Golgi) can remain distinct in the context of a syncytium because of the existence of distinct nuclear pore-associated cytoplasmic domains (Figure 1D-F). Thus the inside-out model predicts that the ER contributes to the separation of distinct domains of transcript accumulation. Furthermore, under the model one would expect there to be a gradient of ER-dependent compartmentalization within syncytia: more compartmentalized close to nuclei due to the presence of pore-associated bleb domains and more continuous nearer the plasma membrane. In a system like the early fly syncytial blastoderm [204,205], this might explain the free diffusion of bicoid mRNA through the peripheral cytoplasm and the differentiation of individual nuclei.

### A role for nuclear pore-associated domains in cell polarity

The inside-out model states that when rates of compartment fusion are low, each nuclear pore communicates to a small portion of local cytoplasm. This has the potential to enable the local translation of transcripts emerging from a pore. This is similar to the ‘gene gating hypothesis’ [206], which proposed that the position of chromosome domains within the nucleus directed the export of locally coded mRNAs through specific pores. While few data exist to support this mechanism, Piwi-interacting RNAs are locally exported from nuclei, leading to their accumulation at specific sites at the nuclear periphery [207]. Moreover, in polarized neurons, RNPs are compartmentalized into nuclear blebs bound by the inner nuclear membrane, like those predicted by the model [108]. We speculate, therefore, that the establishment of subcellular polarity will often involve a role for targeted transport of mRNA through particular nuclear pores to their associated cytoplasmic domains. This might help explain the stereotypical morphology and compartmentalization of some protists and the extraordinarily high proportion of transcripts that have a distinctly polarized cytoplasmic localization in animals cells [208].

### Novel nuclear-associated functions for eukaryotic ESCRTIII

Under the inside-out model, the eukaryotic cell nucleus is homologous to the archaeal cell membrane. This accords with evidence that ESCRTIII is involved in scission of extracellular protrusions in Archaea [54,55] and that it plays a topologically equivalent role (under an inside-out interpretation) in the formation of virus-induced nuclear envelope blebs [209]. Following from this, and given the role of ESCRTIII in cell division in some Archaea [56-58], the model predicts that ESCRTIII will be found to play an important role in remodeling the nuclear envelope at division, especially during closed nuclear divisions, a process that is, as yet, poorly understood [159].

## Conclusions

As described in the preceding sections, the inside-out model can explain the essential features of modern eukaryotes. In the course of describing the model we have pointed to various sources of evidence that support the inside-out model. These can be organized into three broad categories: characteristic features of eukaryotes that are explained parsimoniously by the model; unusual features of eukaryotic cells that are explained by the inside-out model but otherwise seem enigmatic; and direct phylogenetic evidence supporting the inside-out model over outside-in alternatives. We will review these three classes of evidence in turn.

The principle of parsimony states that we should favor models that explain observations while drawing on the fewest *ad hoc* assumptions. The inside-out model is parsimonious in that, simply by assuming that the cytoplasm was formed from extracellular blebs, it can explain diverse features of modern eukaryotic cell organization. For example the model explains the existence of a nuclear compartment that lacks any internal membrane-bound organelles, why typical eukaryotic cells are much larger than most prokaryotes yet have a nucleus similar in size to many eocytes, why there is a double nuclear membrane whose periplasmic space is continuous with ER, why protein N-glycosylation is initiated in the nuclear envelope, and why the ER tends to be continuous with the nuclear envelope. Outside-in models account for the nuclear-ER connection, but new assumptions need to be added to account for ER continuity, for the lack of similarity between the S-layer and eukaryotic cell walls, for N-linked glycosylation machinery having been relocalized from the plasma membrane (S-layer) to the ER, for the fusion of vesicles to form a single nucleus that lacks functional ribosomes, and so on. While these data come far short of ruling out the outside-in view, we believe that the inside-out model offers a simpler explanation for these data.

The second class of evidence comprises assorted quirky features of eukaryotes that would not be predicted under conventional models for the origin of eukaryotes, yet seem to arise quite simply from the inside-out model. Thus, the inside-out model naturally explains why ER tends to show high levels of continuity, even in multinucleate syncytia, where nuclei achieve high degrees of autonomy. Likewise, the model explains the close functional connections between ER and mitochondria and the important roles both organelles play in lipid synthesis. And, as one final example, the inside-out model provides a logical explanation for the presence of phosphoinositides in the nucleus and their role in regulating mRNA processing.

The third class of evidence involves inferences drawn from phylogenetic analyses of eukaryotic gene families. Phylogenetic analysis of the Ras GTPase superfamily has been used to argue that secretion and exocytosis evolved before endocytosis [173] (this conclusion is, however, uncertain given the phylogenetic tree obtained). Such an order of evolution is predicted by the inside-out model, not the outside-in model. Stronger evidence in support of our theory arises from phylogenomic studies that identify α-proteobacteria, and hence mitochondria, as the source of eukaryotic lipid biosynthesis and transport genes [33]. This conclusion is further bolstered by phylogenetic results supporting the eocyte hypothesis [26,27,59], because this tree topology makes it even less probable that eukaryotic lipids were vertically inherited from the last common ancestor of Bacteria and Eukarya. If bacterial lipids were acquired from mitochondria, then it seems almost certain that phagocytosis could not have been the means by which a close symbiosis was established between mitochondria and their eukaryotic hosts. This follows because the acquisition of bacterial lipids would have been a pre-requisite for the dynamic changes in membrane structure required for phagocytosis. The inside-out model is unique among competing theories in allowing close symbiosis of mitochondria and their eukaryotic hosts to be established without phagocytosis, minimizing the number of membrane-interacting proteins affected by a changeover in membrane chemistry. Thus, phylogenetic analysis of lipid biosynthesis genes provides compelling support for the inside-out model over alternatives that rely on one or more early phagocytic events.

In addition to the diverse lines of evidence that are compatible with the inside-out theory, it is worth highlighting that the inside-out model involves a simple series of steps, each of which draws upon plausible ecological and selective drivers. In the early stages, selection favors closer and more extensive physical contact between the host and its ectosymbiotic proto-mitochondria to improve material exchange. Bleb growth and the elaboration of the ER would then have been favored by protecting the mitochondrial flora from being lost to the environment and by protecting ectosymbionts from parasites. In addition, elaboration of the ER would have facilitated the development of a much more sophisticated secretory system that allowed the stepwise processing of proteins prior to export. Movement of mitochondria into the cytoplasm would have eliminated an intervening membrane, further enhancing metabolic exchange. And, finally, formation of a complete plasma membrane would have been advantageous because it closed-off the ER from environmental challenges, such as pathogenic bacteria or viruses, and enabled the complete control of protein modification during secretion.

The inside-out model does not just offer an alternative historical narrative for the origin of eukaryotes. It also provides a new heuristic framework for interpreting the cell biology of contemporary eukaryotic cells. For example, the inside-out model provides an explanation for how nuclei can retain functional autonomy within a syncytium: each nuclear pore of each nucleus can be seen as exerting control over a local, ER-bounded cytoplasmic domain. Indeed, the prevalence of syncytia in eukaryotes, and the difficulties of explaining them using prevailing models, is an argument for the need for a new cell theory to better accommodate cells in which multiple nuclei share a single plasma membrane [15,161]. The inside-out model provides such a framework.

Evolutionary theories dealing with events that occurred billions of years ago are difficult to test. However, there are abundant lines of evidence that could be used in the future to evaluate the inside-out model. This is because the inside-out and outside-in models make strikingly different predictions about the structural homologies of prokaryotic and eukaryotic cells (Table 1) and differ greatly in the order of events through which the endomembrane system evolved (Figure 6). Furthermore, being well constrained, the inside-out model makes a number of specific and testable predictions (Table 2). For example, the model predicts that phylogenomic data will establish that COPII-like coatomers are derived from structural components of the nuclear pore, rather than the reverse; that new nuclear pores will be inserted via outward folding of the inner nuclear membrane mediated by nucleoporins, followed by fusion with overlying membranes; that seven-blade β-propeller proteins function to stabilize the bases of plasma membrane protrusions in archaea; that homologs of SUN proteins may function to tether the cell membrane to the archaeal cell wall; and that there is cellular machinery that functions to promote cytoplasmic continuity in modern cells, which is active both in the late stages of nuclear pore insertion and in the generation of ER fenestrae.

In light of the numerous observations that could be used to discriminate the inside-out and outside-in models, we can be hopeful that empirical tests in the coming years will result in clear support or rejection of this model. Even if the hypothesis or elements of it are refuted, we are optimistic that the effort to evaluate it will spawn new cell biological discoveries and, in so doing, improve our understanding of the biology of archaeal and eukaryotic cells as they grow and divide.
